# Draft Genome Sequences of Four Strains of Plant Growth-Promoting Bacteria Associated with Maize Rhizosphere

**DOI:** 10.1128/mra.00730-22

**Published:** 2022-10-18

**Authors:** Olubukola Oluranti Babalola, Nadège Adoukè Agbodjato, Ayansina Segun Ayangbenro, Adolphe Adjanohoun, Lamine Baba-Moussa

**Affiliations:** a Food Security and Safety Focus Area, Faculty of Natural and Agricultural Sciences, North-West University, Mmabatho, South Africa; b Laboratoire de Biologie et de Typage Moléculaire en Microbiologie, Faculté des Sciences et Techiques, Université d’Abomey-Calavi, Calavi, Benin; c Institut National des Recherches Agricoles du Bénin, Calavi, Benin; University of Rochester School of Medicine and Dentistry

## Abstract

This study presents the draft genome sequences of four strains of rhizobacteria, namely, Bacillus cereus ADO11, Stenotrophomonas maltophilia NAA11, Acinetobacter pittii LAM11, and Serratia marcescens NSA15, which were isolated from maize soils and have the ability to stimulate plant growth. The genome assembly sizes for the strains were 4,476,462 bp, 4,731,402 bp, 4,080,875 bp and 4,959,744 bp, respectively.

## ANNOUNCEMENT

The rhizosphere supports the development and activity of an immense and diverse microbial community, including microorganisms capable of promoting plant growth (1, 2). The bacteria present in this rhizosphere, known as plant growth-promoting rhizobacteria (PGPR), have the ability to promote growth by converting several essential nutrients that are not available to plants into available forms. These multiple activities of PGPR improve soil structure, health, fertility, and function, directly or indirectly promoting plant growth in normal or stressed environments (3). The Bacillus cereus ADO11, Stenotrophomonas maltophilia NAA11, and Acinetobacter pittii LAM11 strains were obtained from rhizospheric maize soils in the southern Benin agroecological zone between 6°30′N and 6°45′N and between 1°35′E and 2°45′E. The Serratia marcescens NSA15 strain was obtained from rhizospheric maize soils in the north Benin agroecological zone. To obtain samples, maize plant roots were cut, with the adhering soil, and mixed in a bucket. The soils of the maize rhizosphere were carefully placed in sterile bags in a cooler containing ice accumulators, transported, and stored at 4°C for 72 h in the Laboratoire de Biologie et de Typage Moléculaire en Microbiologie of the Université d'Abomey-Calavi, Benin for analyses. The soil samples were serially diluted by decimal dilution according to the method described by Speck (4). Isolation of strains was performed on nutrient agar at 37°C for 24 to 48 h. Biochemical and enzymatic tests confirmed phenotypic identifications. The strains were purified and stored at −20°C in Mueller-Hinton broth with 10% glycerol. Bacterial DNA from the four strains was extracted at the North-West University Microbial Biotech Laboratory from a 24-h culture at 37°C on nutrient agar using a Miniprep Quick-DNA kit specific for bacteria or fungi (Zymo Research, Irvine, CA, USA), following the manufacturer’s instructions. The concentration of the extracted DNA was measured using a NanoDrop spectrophotometer (Thermo Fisher Scientific, USA), while the quality of the DNA was assessed on a 2% agarose gel. DNA libraries were generated using a NEBNext Ultra II DNA library preparation kit (catalog number E7645; New England Biolabs). They were then sequenced with a NovaSeq paired-end 150-bp sequencing strategy using the Illumina NovaSeq 6000 platform at Novogen (HK) Co. Ltd. (Singapore, Hong Kong). Sequences were analyzed on the KBase platform (5). Read quality was assessed using FastQC v0.11.5 (6), while removal of sequence adapters and low-quality reads was performed with Trimmomatic v0.36 (7), and reads were assembled with SPAdes v3.13.0 (8).

The final draft genomes for strains ADO11, NAA11, LAM 11, and NSA15 were 4,476,462 bp, 4,731,402 bp, 4,080,875 bp and 4,959,744 bp, respectively, with mean coverage values of 290×, 198×, 115×, and 135×; the total numbers of reads generated for each strain were 8,734,440 reads, 9,861,022 reads, 3,184,574 reads, and 4,510,380 reads, respectively. Gene annotation and prediction were performed using the NCBI Prokaryotic Genome Annotation Pipeline (PGAP) (9). The characteristics of the genomes are summarized in Table 1. All analyses were performed using default parameters. Secondary metabolites were determined with antiSMASH v6.0.0 (10). This strategy identified genes responsible for plant hormone production, transcriptional regulators, transport proteins, and nitrogen fixation, all of which play crucial roles in plants and promote their growth and development (11, 12).

**TABLE 1:** Whole-genome sequencing characteristics of B. cereus ADO11, S. maltophilia NAA11, A. pittii LAM11, and S. marcescens NSA15

Parameter	Data for strain:			
	Bacillus cereus ADO11	Stenotrophomonas maltophilia NAA11	Acinetobacter pittii LAM11	Serratia marcescens NSA15
Genome size (bp)	4,476,462	4,731,402	4,080,875	4,959,744
G+C content (%)	35.4	66.42	38.8	59.8
No. of contigs	28	141	89	56
Genome coverage (×)	290.0	198.0	115.0	135.0
*N*_50_ (bp)	296,930	54,168	88,379	164,348
*L*_50_	4	25	14	11
No. of genes (total)	4,616	4,420	3,977	4,731
No. of proteins	4,467	4,300	3,771	4,629
No. of rRNAs	11	1	5	4
No. of tRNAs	37	67	58	55
No. of other RNAs	5	4	4	12
No. of pseudogenes	96	48	139	33
BioProject accession no.	PRJNA750908	PRJNA751730	PRJNA751219	PRJNA754119
SRA accession no.	SRX11612109	SRX11634702	SRX11616698	SRX11727062
GenBank accession no.	JAIRCN000000000.1	JAIUDP000000000.1	JAMQVB000000000.1	JAMQIW000000000.1

### Data availability.

This whole-genome sequencing shotgun project and associated data have been deposited in DDBJ/ENA/GenBank under the accession numbers listed in Table 1. The GenBank accession numbers are JAIRCN000000000 (ADO11), JAIUDP000000000 (NAA11), JAMQVB000000000 (LAM11), and JAMQIW000000000 (NSA15). The versions described in this paper are the first versions, JAIRCN000000000.1 (ADO11), JAIUDP000000000.1 (NAA11), JAMQVB000000000.1 (LAM11), and JAMQIW000000000.1 (NSA15).
